# Multivariate Analysis of Prognostic Biomarkers in Surgically Treated Endometrial Cancer

**DOI:** 10.1371/journal.pone.0130640

**Published:** 2015-06-24

**Authors:** Jianpei Li, Jianhua Lin, Yaoling Luo, Miaohuan Kuang, Yijun Liu

**Affiliations:** Department of Clinical laboratory, State Key Laboratory of Oncology in South China/Cancer Center of Sun Yat-sen University, Guangzhou, Guangdong province, PR China; Istituto dei tumori Fondazione Pascale, ITALY

## Abstract

**Objective:**

The aim of this study was to identify biomarkers with prognostic value in the setting of surgically treated endometrial cancer.

**Methods:**

Medical data for 282 patients with surgically treated endometrial cancer were reviewed retrospectively. Preoperative concentrations of six serum biomarkers (CA125, CA15-3, C-reactive protein [CRP], D-dimer [D-D], platelet-to-lymphocyte ratio [PLR], and neutrophil-to-lymphocyte ratio [NLR]) were analysed to determine potential associations with clinicopathologic characteristics and to assess prognostic values separately via Kaplan-Meier method and multivariate Cox regression.

**Results:**

In univariate analyses, the 5-year overall survival (OS) rate was 86.5% for a maximum follow-up period of 75 months. High concentrations of CA125, CA15-3, CRP, D-D, PLR, and NLR each proved significantly predictive of poor survival (log-rank test, P<0.01). CRP and D-D were identified as independent prognosticators, using a Cox regression model. Study patients were then stratified (based on combined independent risk factors) into three tiers (P<0.001), marked by 5-year OS rates of 92.1%, 78.4%, and 33.3%.

**Conclusions:**

All serum biomarkers assessed (CA125, CA15-3, CRP, D-D, PLR, and NLR) proved to be valid prognostic indices of surgically treated endometrial cancer. A novel prognostic grouping system, incorporating independent risk factors (CRP and D-D Concentrations), may have merit in assessing these patients preoperatively, providing a biologic basis for improved clinical staging.

## Introduction

Endometrial carcinoma is the most common gynaecologic malignancy [1, 2], accounting for approximately 288,000 new diagnoses and 50,327 deaths worldwide each year [3]. A clinical staging system developed by the International Federation of Gynecology and Obstetrics (FIGO) is currently used to guide surgical management and to predict outcome among patients with endometrial cancer. However, same-stage patients often experience substantially different clinical courses [1, 4]. To address this discrepancy, many investigators have pursued multivariate analysis of tumour attributes to delineate prognostic factors, such as pelvic lymph node metastases (LNM), tumour size (TS), cervical stromal invasion, lymphatic vascular space involvement (LVSI), histologic subtypes, and more [4–6]. Unfortunately, preoperative evaluations generally call for fractional curettage, transvaginal sonography, magnetic resonance imaging, or hysteroscopic assessment, which are invasive, costly, and time-consuming [1, 7–9].

Recently, novel biomarkers (especially those circulating in blood) have been increasingly targeted to predict the course of endometrial cancer. The latter include serum tumour markers (e.g., CA125, HE4, CA15-3) [10, 11], indices of systemic inflammation (e.g., C-reactive protein [CRP], neutrophil-to-lymphocyte ratio [NLR], and platelet-to-lymphocyte ratio [PLR]) [12, 13], and factors implicated in venous thromboembolism (VTE) (e.g., thrombin-antithrombin III complex and D-dimer [D-D]) [14]. Such markers are readily monitored through relatively noninvasive means.

For this study, predictive values of six circulating biomarkers (CA125, CA15-3, CRP, D-D, PLR, and NLR) were addressed via Kaplan-Meier method and multivariate Cox regression. We then used two independent risk factors to develop a prognostic grouping system, thus identifying meaningful prognostic subsets of the study population (as opposed to clinical staging).

## Materials and Methods

### Patients

A retrospective review was conducted, analysing data on 282 patients (age range, 21–76 years; median, 53 years) subjected to surgery at Sun Yat-Sen University Cancer Center (SYSUCC; Guangzhou, China) as primary treatment of endometrial cancer between September 2007 and June 2009. Insufficient data, non-surgical treatment, secondary malignancies, and haematologic diseases were grounds for exclusion.

Each diagnosis of endometrial cancer was based on curetted tissue. Classified by differences in histology and molecular characteristics, endometrial carcinoma has been generally distinguished as Types I (mainly endometrioid) and II (non-endometrioid) as suggested by Bokhman and subsequent researchers.[15–18] For histologic grading of tumours, WHO classification was used. All patients were clinically staged according to FIGO guidelines (2009). Patients underwent total abdominal hysterectomy only (195 cases), or total abdominal hysterectomy plus total abdominal hysterectomy bilateral salpingoo-ophorectomy, and systemic pelvic lymphadenectomy (87 cases); among these 282 surgeries, 110 cases were with and 172 cases withoutpara-aortic lymph node dissection. Radical hysterectomy was performed in instances of suspected cervical stromal involvement. Both common iliac and obturator nodes (above obturator nerve) were included in pelvic lymphadenectomies. Adjuvant chemotherapy was administered postoperatively at the discretion of gynaecologic oncologists overseeing patients with lymph node metastases, parametrial invasion, and positive or close surgical margins. Information about the expression of serum biomarkers is stratified according to endometrial cancer stage, grade, type and patient's age, shown in Table 1.

**Table 1:** The relationship between clinicopathological characteristics and serum biomarkers concentrations. (Mean and 95% CI).

	n	**CA125**	*P v*		CA15-3	*P v*	CRP	*P v*	D-D	*P v*	PLR	*P v*	NLR	*P v*
		(U/ml)			(U/ml)		(mg/l)		(mg/l)					
**Age**														
**<50**	98	23.82		0.491	12.92	0.336	1.13	0.002	0.55	0.286	147.64	0.001	2	0.257
		(14.08–46.81)			(9.14–19.89)		(0.46–2.89)		(0.40–0.90)		(113.36–208.92)		(1.52–2.74)	
**≥50**	184	21.11			12.19		2.17		0.6		125.1		1.83	
		(14.07–39.90)			(8.88–17.71)		(0.72–6.19)		(0.40–1.00)		(99.13–165.96)		(1.38–2.69)	
**Type**														
**Ⅰ**	254	132.18		0.708	21.67	0.787	6.42	<0.001	1.34	0.349	148.87	0.008	2.29	<0.001
		(41.11–223.23)			(14.04–29.31)		(3.59–9.34)		(0.45–2.23)		(140.15–157.96)		(2.11–2.46)	
**Ⅱ**	28	79.55			24.91		33.62		2.65		191.45		3.88	
		(35.68–123.28)			(7.81–42.01)		(13.79–53.46)		(1.22–4.08)		(137.34–245.57)		(2.51–5.25)	
**FiGO**														
**Ⅰ**	152	18.73		<0.001	10.97	<0.001	1.33	0.003	0.5	<0.001	126.47	0.365	1.8	0.054
		(13.31–25.62)			(7.80–15.59)		(0.54–4.20)		(0.30–0.73)		(106.33–167.91)		(1.37–2.46)	
**Ⅱ**	58	25.53			12.37		1.33		0.6		129.33		1.9	
		(15.93–46.99)			(9.37–19.35)		(0.58–4.70)		(0.45–0.90)		(99.03–200.59)		(1.46–2.88)	
**Ⅲ**	61	44.41			15.99		3.07		0.9		139.07		2.08	
		(20.65–72.30)			(10.84–29.3)		(0.82–10.43)		(0.50–1.70)		(104.76–185.46)		(1.52–3.47)	
**Ⅳ**	11	93.19			27.93		3.17		1.4		155		2.84	
		(57.93–386.90)			(10.27–43.3)		(2.28–29.68)		(0.65–2.45)		(111.20–255.14)		(1.60–3.20)	
**Grade**														
**G1**	69	17.75		0.001	11.16	0.003	1.11	0.011	0.5	<0.001	145	0.124	2.1	0.126
		(13.22–23.01)			(7.45–15.21)		(0.45–3.27)		(0.30–0.90)		(114.94–195.72)		(1.30–2.55)	
**G2**	145	25.08			13.07		1.7		0.55		129.5		1.76	
		(13.73–44.66)			(9.25–19.01)		(0.68–4.83)		(0.40–0.80)		(101.95–167.49)		(1.44–2.60)	
**G3**	68	26.07			13.82		2.67		0.9		122.73		2.18	
		(15.75–62.43)			(9.70–24.82)		(0.81–12.19)		(0.50–1.70)		(101.09–205.83)		(1.49–3.32)	
**Metas.**														
**Yes**	14	20.51		0.689	14.26	0.97	4.96	0.019	1	0.021	138.71	0.522	2.3	0.231
		(11.86–59.16)			(7.28–23.54)		(1.05–48.76)		(0.65–1.73)		(108.04–243.000)		(1.40–4.63)	
**No**	268	21.54			12.47		1.49		0.6		130		1.89	
		(14.09–44.40)			(9.12–18.99)		(0.58–4.94)		(0.40–0.90)		(105.14–177.72)		(1.43–2.67)	
**Recur.**														
**Yes**	12	17.37		0.994	11.38	0.808	11.14	0.001	0.7	0.081	140.35	0.384	2.9	0.031
		(9.62–274.60)			(7.55–50.66)		(2.38–63.18)		(0.50–4.10)		(92.98–332.45)		(1.85–5.09)	
**No**	270	21.47			12.5		1.47		0.6		130.25		1.88	
		(14.13–41.76)			(9.10–18.83)		(0.59–4.83)		(0.40–1.00)		(105.94–177.31)		(1.42–2.67)	

*P-v*: p value; Type: types of endometrial carcinoma; FIGO: the FIGO stage; Grade: Histopathological grade; D-D: D dimer; PLR: platelet to lymphocyte ratio; NLR: neutrophil to lymphocyte ratio; Metas.: metastasis; Recur.: recurrence

### Methods

All data on six blood biomarkers (CA125, CA15-3, CRP, D-D, PLR, and NLR) were collected from preoperative medical records of patients studied. Peripheral blood samples drawn from patients less than 2 weeks prior to surgery were analysed in the SYSUCC clinical laboratory. A Modular Analytics E170 immunoassay unit (Roche Diagnostics, Germany) was used to determine concentrations of tumour markers (CA125, CA15-3), a Hitachi 7600 automated chemistry analyser (Hitachi Co, Japan) was used to determine CRP concentrations, a latex agglutination assay for D-D concentrations (Sekisui Medical Co, Ltd, Japan) and a Sysmex XE-5000 system (Sysmex Co, Japan) for blood cell counts. NLR (absolute neutrophil count divided by absolute lymphocyte count) and PLR (absolute platelet count divided by absolute lymphocyte count) were both computed. Patients were separately divided into two groups with high or low concentrations by the cut-points (CA125 ≥35 U/ml, CA15-3 ≥25 U/ml, CRP, ≥8.2 mg/L, D-D ≥1.5 mg/l, PLR ≥250, and NLR ≥4.68) as recommended by reagent manufacturers or cited other authors [12, 13, 19–23].

The interrelationships of clinicopathologic features, serum biomarker concentrations, and cancer prognosis were analysed. Each patient returned on a semi-annual basis for clinical follow-up visits by a specified division of SYSUCC, and follow-up information accrued until death or a maximum of 75 months (mean: 51.2 months, range: 0.3–75.8 months) at the time of study completion (March 2014).

### Ethical Statement

The study was approved by the Clinical Research Ethics Committee of SYSUCC. Patient records and information were anonymized and de-identified prior to analysis.

### Statistical analysis

Data analysis relied on standard software (SPSS v 17.00; SPSS Inc., Chicago, IL, USA). Survival curves were constructed via Kaplan-Meier method and comparisons were made using the log-rank test. For univariate and multivariate analyses of prognostic factors, Cox proportional hazards regression was applied. Statistical comparisons among the three tiers of patients were achieved through one-way ANOVA, with Student-Newman-Keuls post hoc testing. Student's t-test or Mann-Whitney U-test was used to assess significance of between-groups differences. Statistical significance was set at P<0.05.

## Results

### Relationships between clinicopathologic characteristics and serum biomarker concentrations

A total 282 patients with endometrial cancer who were treated surgically and fully met our criteria were included in the study. Patient demographics, clinicopathologic characteristics, and biomarker concentrations are summarised in Table 1. Higher CRP concentrations (P<0.01) and lower PLR concentrations (P<0.01) were observed in older subjects (≥50 years). In patients with Type II endometrial carcinoma, higher concentrations (P<0.01) of CRP, PLR and NLR were observed. Concentrations of tumour markers (CA125 and CA15-3) rose significantly (P<0.01) as FIGO stage and histopathologic tumour grade worsened. Concentrations of CRP were clearly elevated in patients with tumour metastases or recurrences (Table 1). Median D-D concentration (0.6 mg/l) exceeded the reagent provided normal reference value (<0.5 mg/l); and 34 patients with D-D plasma concentrations >1.5 mg/l were felt to be at risk of silent VTE. Significant elevations of NLR (P<0.01) were confined to patients with recurrent neoplasms.

### Prognostic value of biomarkers in endometrial cancer

**Fig 1:**
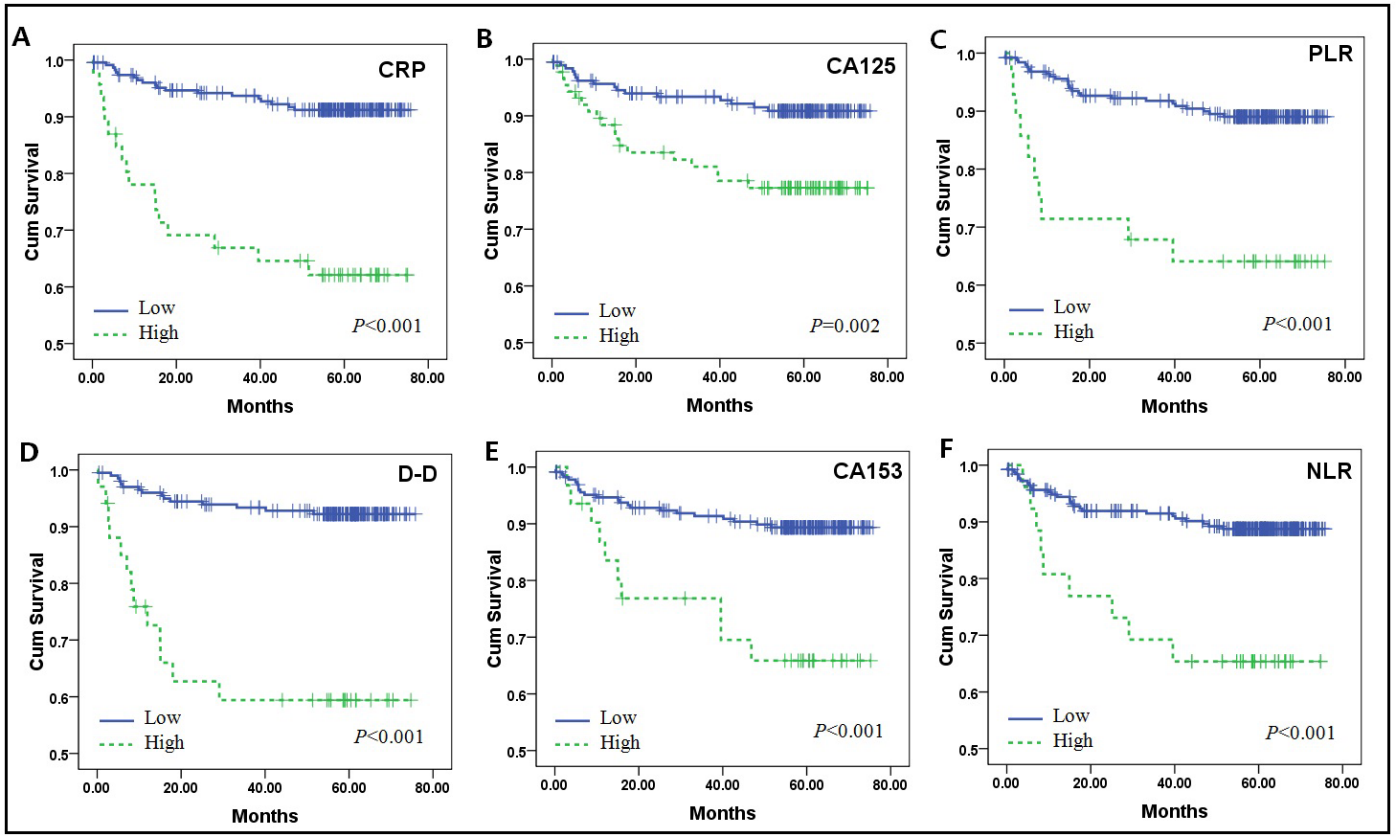
Kaplan-Meier survival estimates for patients with endometrial cancer. Disease-specific survival curves of patients grouped as low and high risk, according to biomarker cut-points (log-rank test used to calculate P-values).

Patient survival curves, assigned by concentrations of biomarkers to high and low groups, were constructed via Kaplan-Meier method and were compared using the log-rank test (Fig 1). Mean survival of the cohort was 67.9 months, with a 5-year overall survival (OS) of 86.5%. As shown in Table 2 and Fig 1, elevations of all biomarkers assessed (CA125, CA15-3, CRP, D-D, PLR, and NLR) were significantly predictive of poor 5-year OS (P<0.01). Among these variables, only CRP and D-D concentrations were identified as independent prognostic variables, based on the Cox proportional hazards model depicted in Table 3. Respective hazard ratios for CRP and D-D were 0.215 (95% CI: 0.084–0.549) and 0.252 (95% CI: 0.095–0.670)

**Table 2:** Univariate analysis of biomarkers predictive of survival time (S.T) and the 5-year overall survival (OS).

	n	S T(month)	5-year OS (%)	*P*-v
		Mean (95%,CI)	Mean±SD	Log-Rank
**Total**	282	67.9(65.4–70.4)	86.5±1.9	
**CRP**				
**low**	232	70.9(68.7–73.1)	91.2±1.9	<0.001
**high**	46	51.8(42.7–60.8)	62.1±7.3	
**D-D**				
**low**	202	71.2(68.9–73.5)	92.2±1.9	<0.001
**high**	34	48.4(37.3–59.6)	59.4±8.7	
**CA125**				
**low**	187	70.6(68.1–73.1)	90.9±2.2	0.002
**high**	88	61.8(56.4–67.3)	77.3±4.6	
**CA15-3**				
**low**	229	69.6(67.1–72.0)	89.4±21	<0.001
**high**	31	56.4(46.5–66.3)	65.9±8.8	
**PLR**				
**low**	254	69.6(67.3–71.9)	89.5±2.0	<0.001
**high**	28	52.1(40.3–63.8)	64.1±9.1	
**NLR**				
**low**	256	69.3(66.9–71.7)	89.2±2.0	<0.001
**high**	26	54.3(43.2–65.4)	65.4±9.3	

Cut-off values for each biomarker: CA125 35U/ml; CA15-3 25U/ml; CRP 8.2mg/L; D-D 1.5mg/l; PLR 250, NLR 4.68.

**Table 3:** Multivariate Cox regression analyzes of serum biomarkers prognostic factors for surgical patients with endometrial cancer.

	Coefficient	SE	*P-v*	HR	95.0% CI for HR
**CRP**	-1.536	0.477	0.001	0.215	0.084–0.549
**DD**	-1.376	0.498	0.006	0.252	0.095–0.670
**CA125**	0.071	0.526	0.893	1.073	0.383–3.009
**CA15-3**	-0.621	0.540	0.250	0.537	0.186–1.550
**PLR**	-0.007	0.621	0.991	0.993	0.294–3.357
**NLR**	0.832	0.622	0.181	2.298	0.679–7.781

SE: standard error; *P-v*: P value; HR: hazard ratios; CI: confidence interval.

### A novel prognostic grouping system combining independent risk factors

**Fig 2:**
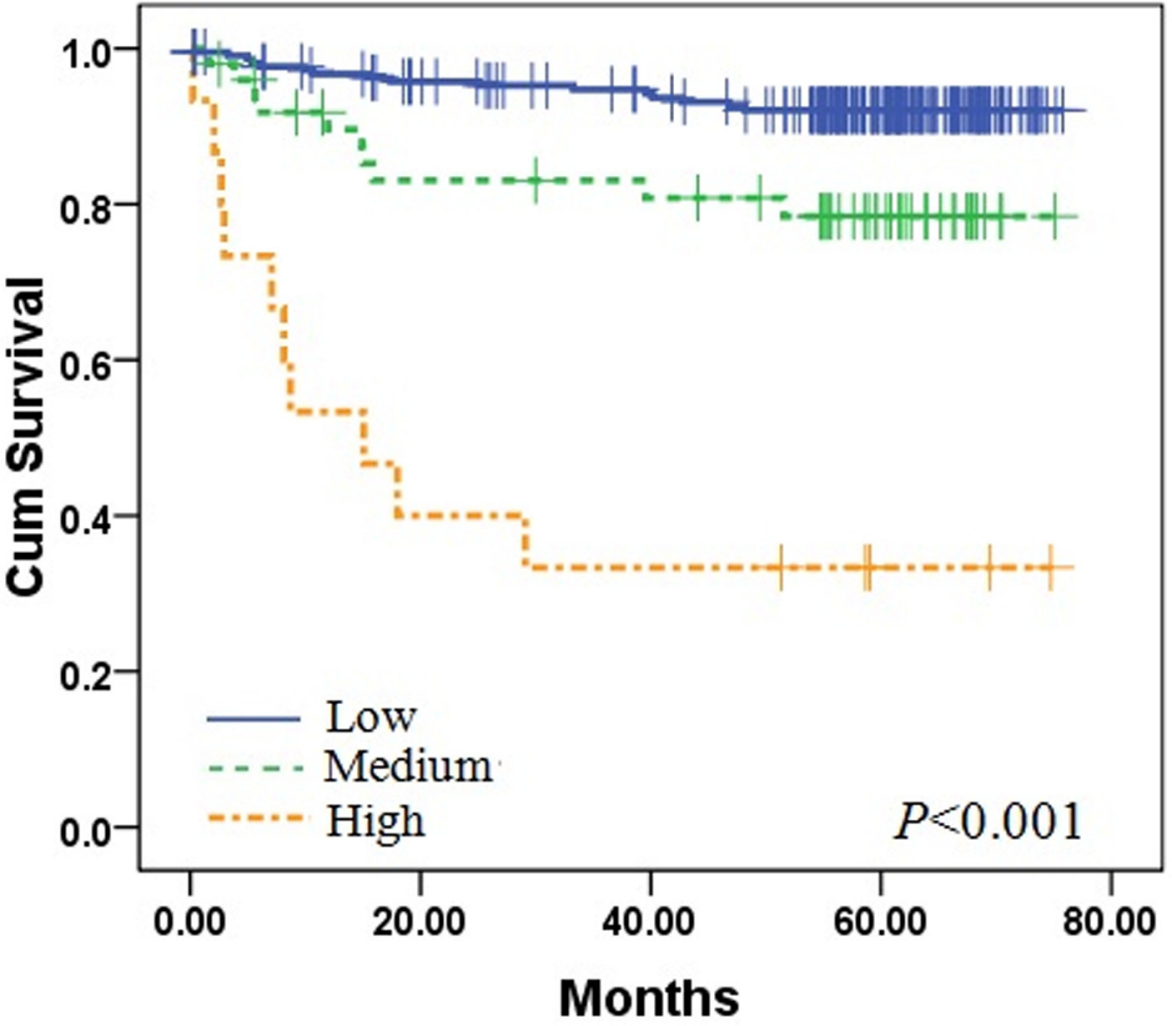
Three-tiered stratification of patients with endometrial cancer, incorporating two independent prognostic variables (CRP and D-D Concentrations). Survival in low-risk (CRP <8.2 mg/l and D-D <1.5 mg/l), medium-risk (CRP ≥8.2 mg/l, or D-D ≥1.5 mg/l), and high-risk (CRP ≥8.2 mg/l and D-D ≥1.5 mg/l) groups differed significantly (log-rank test used to calculate P-values).

As independent risk factors in this analysis, CRP and D-D concentrations prompted a new prognostic hierarchy where stipulated subsets better reflected actual outcomes of patients with endometrial cancer. Patients were assigned as follows: low risk (CRP <8.2 mg/l and D-D <1.5 mg/l), medium risk (CRP ≥8.2 mg/l, or D-D≥1.5 mg/l), and high risk (CRP ≥8.2 mg/l and D-D≥1.5 mg/l). Patient survival differed significantly by group (Fig 2; P<0.001), with mean survival times as follows: low risk, 71.5 months (95% CI: 69.6–73.6; n = 217); medium risk, 62.6 months (95% CI: 55.5–69.7; n = 50); and high risk, 31.2 months (95% CI:15.2–47.2; n = 15). Respective 5-year OS rates were 92.1%±1.9, 78.4%±6.1, and 33.3%±12.2.

## Discussion

In the course of this study, the prognostic values of six blood biomarkers in patients with endometrial cancer were evaluated. To our knowledge, this is the first effort where CRP and D-D concentrations have been identified as independent prognostic factors in endometrial cancer.

Although the 5-year survival rates of endometrial cancer are favourable in general [24], some patients have poor outcomes. Clinicopathologic characteristics of endometrial cancer, including patient age, histologic type/grade, clinical stage, lymphatic vascular space invasion, and other parameters, have been well-studied previously [25–27]. Current efforts increasingly have targeted molecular biomarkers that may advance the histologic classification of tumours, improve our ability to identify more aggressive cancers [28–32], and potentially enable staging revisions, resulting in more personalized therapy and better clinical outcomes. In this study, we successfully defined a poor prognosis group by incorporating the above independent risk factors in a new system of patient stratification (Fig 2).

As the goal of this investigation, relationships between concentrations of certain biomarkers and clinicopathologic characteristics of endometrial cancer were analysed. Patient age clearly is an important risk factor here. In the 15% of patients diagnosed with endometrial cancer before the age of 50 [33], the 5-year survival rate is nearly 96.3% [25]. Our evidence shows that both CRP concentration and PLR are significantly aligned with patient age. In addition, FIGO surgical staging (recommended as a formal step in initial treatment)[1] showed significant associations with CA125, CA15-3, CRP, and D-D Concentrations in our study, these indicate that they are closely related with tumour pathophysiology. There have been many reports on the risk factors and molecular markers associated with Type I and Type II endometrial cancer [18, 34], in this study we found that CRP, PLR and NLR in the blood were significantly associated with histologic subtypes of endometrial cancer.(Table 1), These differences may be the result of more severe system inflammation in Type II endometrial cancer. We also found that the histologic grades of tumours correlated with CA125, CA15-3, CRP, and D-D Concentrations, however, only CRP and D-D elevations were seen in patients with metastatic or recurrent tumours (Table 1). The above findings suggest that these risk factors are all interrelated.

We further analysed the prognostic value of the panel of biomarkers via Kaplan-Meier method and Cox proportional hazards regression. In univariate analyses, all six biomarkers correlated significantly with survival time (Fig 1), but in multivariate analyses, only CRP and D-D concentrations were independently linked with endometrial cancer prognosis. Elevated serum CA125 concentrations carry a number of connotations in patients with endometrial cancer, implying advanced age and tumour stage, shortened survival, and extrauterine spread, among other features [22, 23]. Consistent with previous findings, high serum CA125 concentration (CA125 ≥35 U/ml) was also a risk factor for poor prognosis (5-year OS,77.3%±4.6) on this occasion. Although the CA15-3 biomarker was similarly indicative of poor prognosis in this cohort, no highly significant association with shortened survival was evident in multivariate analysesas previously found [35]; this lack of significance may be due to its poor specificity for endometrial cancer.

Chronic inflammation may well play a central role in development of endometrial cancer [36]. As CRP is the prevailing index of inflammation [37], the CRP concentration elevations seemingly validate a link between obesity and endometrial cancer [29, 30]. We have shown that 5-year OS rates differed significantly (P<0.01) in patients with low (91.2%) and high (14.4%) CRP concentration. In addition, the considerable significance of CRP concentration elevation was sustained in multivariate analyses, coupled with a greater hazard ratio (Table 3). PLR and NLR are also indices of systemic inflammation, which recently have been explored as prognosticators in various cancers (pancreatic, colorectal, ovarian, gastric, and endometrial). However, the prognostic role of PLR in cancer currently remains controversial [12, 13, 32]. In Table 1, PLR showed a significant association with age, and NLR correlated significantly with FIGO stage and tumour recurrence. In terms of survival, both parameters proved significant by univariate analyses but not in the Cox regression model.

High D-D concentration (>1.5 mg/l) is likely a harbinger of VTE [38]. One previous study documented silent or subclinical VTE prior to surgery in at least 10% of patients with endometrial cancer, underscoring that D-D determination should be considered before treating any of these patients [19]. In this study, D-D concentrations were significantly aligned with FIGO stage and histologic tumour grade, as well as with tumour metastasis and recurrence. Understandably, those patients (9.2%) with high D-D concentrations (>1.5 mg/l) had even worse prognoses (Table 2), and D-D concentration was found to be an independent prognosticator via Cox regression model.

Detection of VTE and inflammatory conditions prior to surgical treatment is a very important aspect of therapeutic strategy and postoperative care [1, 19, 39]. In this study, both CRP and D-D concentrations were identified in multivariate analyses as independent prognostic variables for survival in patients with endometrial cancer. Hence, we developed a new prognostic hierarchy for grouping of patients, incorporating CRP and D-D cut-points. Survival of patients postoperatively differed significantly at these three new tiers of patient risk (Fig 2). We feel this is useful tool for preoperative assessment of patients with endometrial cancer. Furthermore, this approach may provide more objective biologic evidence to improve the existing system of clinical staging.

At present, FIGO surgical staging relies on pre- and intraoperative pathologic assessment of tumour grade and depth of invasion, but by some accounts, one-third of grade 1 endometrial cancers are upgraded, and one-third are upstaged once final pathology reports are issued [39, 40]. Serum biomarker determinations may outperform histologic and even imaging studies as a means of tumour assessment, enabling better appreciation of biologic attributes in a more timely fashion. The molecular signatures of cancers provide clues to the cellular microenvironment, offering opportunities for personalised treatment strategies [39].

In patients surgically treated for endometrial cancer, preoperative concentrations of the above six biomarkers studied showed prognostic value. Moreover, CRP and D-D concentrations were proven (via Cox regression model) to be independent indices of prognosis in this setting, prompting the development of a novel patient stratification system.
